# Supplementary figures and images for: Giant magnetoresistive biosensors for real-time quantitative detection of protease activity
Source: Sci Rep. 2020 May 14;10:7941. doi: 10.1038/s41598-020-62910-2 (PMC7224196; doi:10.1038/s41598-020-62910-2)

a)

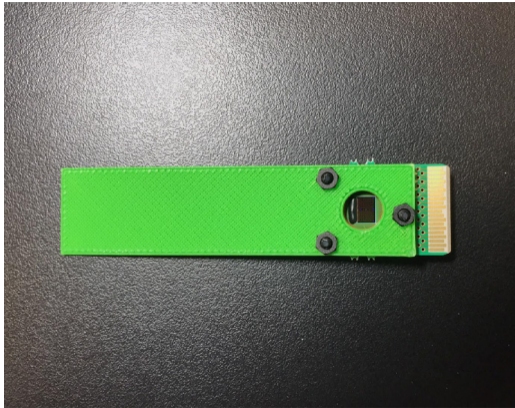

b)

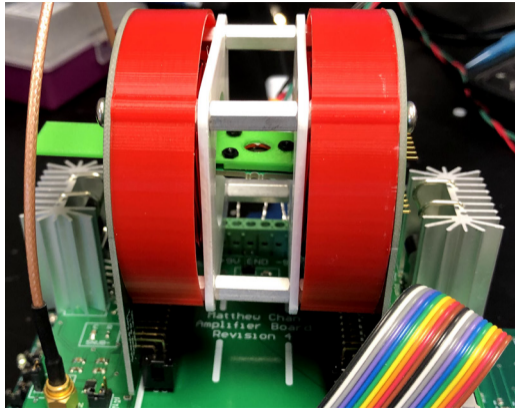

Supplement: Supplementary file 1 — Supplementary information [file 41598_2020_62910_MOESM1_ESM.pdf]
